# Comparative Effects of Traditional Versus Genetically Modified Soybean Oils on Colon Tumorigenesis in Mice

**DOI:** 10.3390/foods11131937

**Published:** 2022-06-29

**Authors:** Maolin Tu, Quancai Sun, Jianan Zhang, Guodong Zhang

**Affiliations:** 1 Department of Food Science, University of Massachusetts, Amherst, MA 01003, USA; tumaolin012@163.com (M.T.); jiananz@email.unc.edu (J.Z.); 2 School of Food Science and Technology, National Engineering Research Center of Seafood, Collaborative Innovation Center of Seafood Deep Processing, Dalian Polytechnic University, Dalian 116034, China; 3 Department of Food Science and Technology, National University of Singapore, Singapore 117542, Singapore; sqctp8@nus.edu.sg; 4 Molecular and Cellular Biology Graduate Program, University of Massachusetts, Amherst, MA 01003, USA

**Keywords:** soybean oil, genetically modified soybean oil, colon cancer

## Abstract

Soybean oil, which has high abundance of linoleic acid (LA, 18:2ω-6), is the most commonly consumed edible oil. Recent studies support that a high dietary intake of LA is linked with increased risks of developing colonic inflammation and colon cancer. Here we studied the effects of the genetically modified Plenish^®^ soybean oil, which has low abundance of LA as well as α-linolenic acid (ALA, 18:3ω-3), on development of azoxymethane (AOM)/dextran sulfate sodium (DSS)-induced colon tumorigenesis in mice. Compared with a diet rich in traditional soybean oil, administration of a diet enriched with the Plenish oil has little impact on AOM/DSS-induced colon tumorigenesis, colonic infiltration of immune cells, expressions of inflammatory genes, and tumor markers. These results suggest that the traditional and the Plenish soybean oils have similar effects on development of AOM/DSS-induced colon cancer in mice.

## 1. Introduction

The consumption of soybean oil has dramatically increased in recent decades: its consumption has risen more than ~47% since 1980 and more than ~1000-fold since 1909 [1]. Today, soybean oil is the most commonly consumed vegetable oil in the U.S. and accounts for ~80% of edible oil consumption in the U.S. [2]. Soybean oil contains a high level of linoleic acid (LA, 18:2ω-6): >50% of the fatty acid in soybean oil is LA [2]. Recent animal studies showed that a high intake of LA exacerbates the development of azoxymethane (AOM)- or *Apc* gene mutation-induced colon tumorigenesis [3,4,5,6,7]. Human studies also support that a high intake of dietary LA is linked with increased risks of developing colon cancer [8,9] and colonic inflammation [10,11,12,13], though there are inconsistent results from human studies which showed that LA does not have adverse effects [14,15,16]. Furthermore, as a polyunsaturated fatty acid (PUFA), LA is prone to non-enzymatic lipid oxidation and is therefore chemically unstable [17]. During food preparation and processing, LA in soybean oil could be oxidized and/or degraded, leading to formation of lipid oxidation-derived compounds, and this is associated with off flavor, shelf-life reduction, and potentially harmful health effects [17,18].

Plenish^®^ soybean oil, which enters the U.S. market in 2014, is a genetically modified soybean oil [19]. The Plenish oil has reduced abundance of LA. The level of LA in traditional soybean oils vs. the Plenish oil is 54.0% vs. 7.3% (see Supplemental Information in Table S1). Besides LA, the Plenish oil has different profiles of other fatty acids: notably, the amount of α-linolenic acid (ALA, 18:3ω-3) in traditional vs. Plenish oils is 6.9% vs. 1.7% (Table S1). Epidemiological and pre-clinical studies support the beneficial effects of ω-3 PUFAs on colonic inflammation and colon carcinogenesis [9,20,21,22,23,24]. Considering that the Plenish oil has reduced content of ω-6-series LA and reduced level of ω-3-series ALA, both of which could be important in colon tumorigenesis, it is intriguing to study its effects on development of colon cancer. In this study, we compared the effects of traditional soybean oil with the genetically modified Plenish oil on development of AOM/dextran sulfate sodium (DSS)-induced colon tumorigenesis in mice.

## 2. Materials and Methods

### 2.1. Animal Experiment

The animal experiment was approved by the Institutional Animal Care and Use Committee of the University of Massachusetts Amherst. C57BL/6 male mice (6 weeks old) were obtained from Charles River (Wilmington, MA, USA) and treated with 10 mg/kg AOM (Sigma-Aldrich, St. Louis, MO, USA) via intraperitoneal injection; one week later, they were given 2% DSS (36–50 kDa, MP Biomedicals, Santa Ana, CA, USA) in drinking water for 7 days. These mice were then divided into two groups (*n* = 11 mice per group) and treated with: (i) control diet (rich in traditional soybean oil) and (ii) Plenish oil-rich diet (rich in Plenish soybean oil). The diet compositions and fatty acid profiles are shown in Table 1 and Table 2. The commercial samples of traditional soybean oil and Plenish soybean oil were obtained as gifts from Corteva Agriscience (Johnston, IA, USA).

The mice were sacrificed at week 9 post the AOM injection. The colon tissue was harvested and cut longitudinally, the intestinal contents were washed off with cold PBS, and the number and size of tumors were counted under a microscope. The mouse body weight was analyzed by comparison with the body weight at t = 0 (the day of AOM injection).

### 2.2. Flow Cytometry Analysis

Distal colon tissues were dissected, washed with cold PBS, and digested with Hank’s-balanced salt solution (HBSS, Lonza, Basel, Switzerland) supplemented with 5 mM EDTA and 1 mM dithiothreitol (DTT) for 2 h at 4 °C. The released cells were filtered through 70 μm cell strainer (BD Biosciences, San Jose, CA, USA) to obtain single cell suspensions, which were stained with anti-CD45, anti-F4/80, and anti-GR-1 antibodies following the manufacturer’s instructions. The stained cells were measured using BD LSRFortessa™ cell analyzer (BD Biosciences, San Jose, CA, USA) and the data were analyzed using FlowJo software (FlowJo LLC, Ashland, OR, USA). In our analysis, leukocytes were identified as CD45^+^ cells, macrophages were identified as CD45^+^ F4/80^+^ cells, and neutrophils were identified as CD45^+^ GR-1^+^ cells.

### 2.3. H&E Staining

Colon tissue was fixed in formalin, embedded in paraffin (Thermo Fisher Scientific, Waltham, MA, USA), cut into 5-µm sections, dewaxed in xylene (Thermo Fisher Scientific), rehydrated through graded ethanol solutions (Pharmco-Aaper, Shelbyville, KY, USA), stained with hematoxylin and eosin (Sigma-Aldrich), and examined using a light microscopy.

### 2.4. qRT-PCR Analysis of Gene Expression

Total RNA was extracted from colon tissues with Trizol reagent (Ambion, Austin, TX, USA). The extracted RNA was reverse transcripted into cDNA with a High Capacity cDNA Reverse Transcription kit (Applied Biosystems, Foster City, CA, USA) following the manufacturer’s instructions. qRT-PCR was performed in a DNA Engine Opticon system (Bio-Rad Laboratories, Hercules, CA, USA) with SYBR-green Master Mix kit (Thermo Fisher Scientific). The primer sequences (Thermo Fisher Scientific) are shown in Table 3. The results of target genes were normalized to glyceraldehyde-3-phosphate dehydrogenase (Gapdh).

### 2.5. Statistical Analysis

All data are expressed as the mean ± standard error of the mean (SEM). Statistical analysis was performed by using Graphpad Prism 6 and *p* < 0.05 was considered statistically significant. For the comparison between two groups, Shapiro–Wilk test was used to verify the normality of data; when data were normally distributed, statistical significance was determined using two-side *t*-test; otherwise, significance was determined by Mann–Whitney test.

## 3. Results

### 3.1. Characterization of Commodity Soybean Oil- or Plenish Soybean Oil-Rich Diets

We treated mice with two completely defined isocaloric diets that had the same level of total fat content (7 wt/wt% of total fat) but contained different dietary fats. The control diet had a fat content of 7 wt% commodity soy oil, and the Plenish oil-rich diet contained 1.5 wt% commodity soy oil and 5.5 wt% Plenish soy oil (see diet composition in Table 1). Based on the fatty acid profiles of the traditional and Plenish soybean oils (Table S1), the control diet contained 54.0% of LA (18:2ω-6), 6.9% ALA (18:3ω-3), and 21.2% OA (18:1ω-9), while the Plenish oil-rich diet contained 17% LA, 2.8% ALA, and 65.6% OA (see fatty acid profiles of diets in Table 2).

### 3.2. Effects of Plenish Soybean Oil on Colon Tumorigenesis

**Figure 1:**
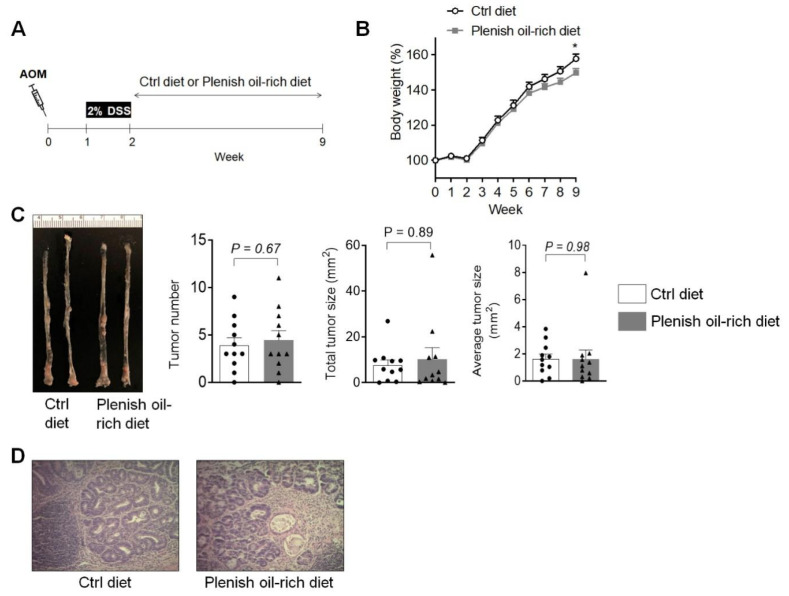
Effects of Plenish soybean oil on AOM/DSS-induced colon tumorigenesis. (**A**) Scheme of the animal experiment. (**B**) Plenish oil-rich diet slightly reduced body weight *(n* = 11 mice per group). (**C**) Plenish oil-rich diet had little impact on AOM/DSS-induced colon tumorigenesis. (**D**) Histology of the colon tumors (*n* = 11 mice per group).

We stimulated C57BL/6 mice with AOM and DSS to induce colon tumorigenesis, then treated the mice with the control diet or Plenish oil-rich diet (see scheme of the animal experiment in Figure 1). After AOM/DSS treatment, the mice treated with Plenish oil-rich diet had slightly reduced body weight: at the end of the experiment (9 weeks post the AOM injection), the body weight of the mice, expressed as relative weight compared with the mouse weight at t = 0, were 157.8 ± 2.7 (mean ± SEM) % vs. 149.7 ± 2.5% (*p* < 0.05) for control group and Plenish oil group, respectively (Figure 1B).

In terms of colon tumorigenesis, we found that the control diet and the Plenish oil-rich diet had similar effects on development of AOM/DSS-induced colon tumorigenesis in mice (Figure 1C,D). The tumor number, total tumor burden, and average tumor size of for control group vs. Plenish oil group was 3.9 ± 0.8 (mean ± SEM) vs. 4.5 ± 1.0 (*p* = 0.67), 7.6 ± 2.3 vs. 10.3 ± 5.0 (*p* = 0.89), and 1.6 ± 0.4 vs. 1.6 ± 0.7 (*p* = 0.98), respectively (Figure 1C).

### 3.3. Effects of Plenish Soybean Oil on Colonic Infiltration of Immune Cells

**Figure 2:**
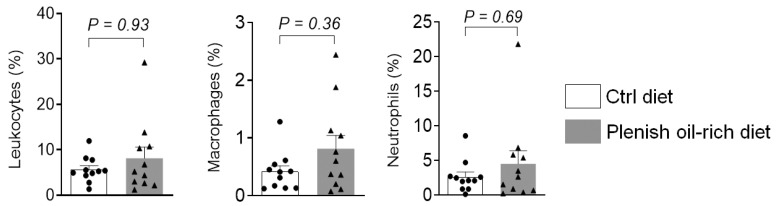
Quantification of immune cells in colon. The results are expressed as mean ± SEM, *n* = 11 mice per group.

We used FACS to quantify immune cell infiltration in colon tissues (Figure 2). Compared with the control diet, treatment with Plenish oil-rich diet did not significantly alter the abundance of leukocytes (CD45^+^), macrophages (CD45^+^ F4/80^+^), or neutrophils (CD45^+^ Gr1^+^) in the colon tissues. This result is in agreement with the colon tumorigenesis data above (Figure 2), further supporting that the Plenish soybean oil has similar effects on development of colon cancer compared with the traditional soybean oil.

### 3.4. Effects of Plenish Soybean Oil on Colonic Expression of Inflammatory Genes

**Figure 3:**
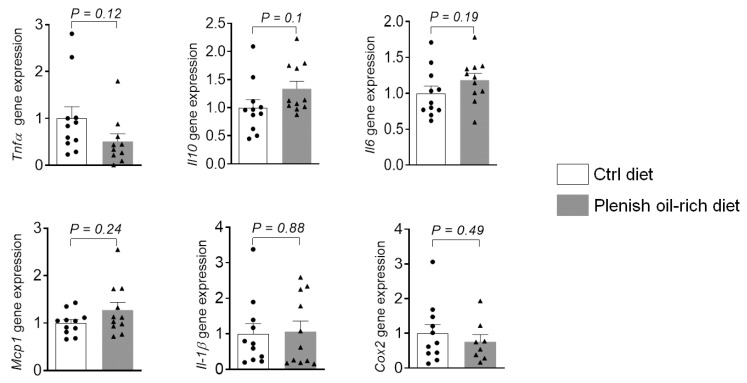
Effects of Plenish soybean oil on colonic expression of inflammatory genes. The results are expressed as mean ± SEM, *n* = 8–11 mice per group.

We also analyzed the expression of inflammatory genes in colon tissues (Figure 3). Compared with the control diet, treatment with Plenish oil-rich diet did not significantly alter the expression of a series of genes that are important in inflammation, including *Tnf-α*, *Il-10*, *Il-6*, *Mcp-1*, *Il-1β*, and *Cox-2* (Figure 3). These genes encode the expression of proteins that are critical for inflammation and colon tumorigenesis [25]. For example, substantial studies have shown that *Cox-2*, which mediates the expression of cyclooxygenase (COX-2), plays critical roles in the pathogenesis of colon cancer [26]. Overall, these results support that Plenish oil-rich diet did not have a significant effect on tumor-associated colonic inflammation compared with the control diet.

### 3.5. Effects of Plenish Soybean Oil on Colonic Expression of Tumor Markers

**Figure 4:**
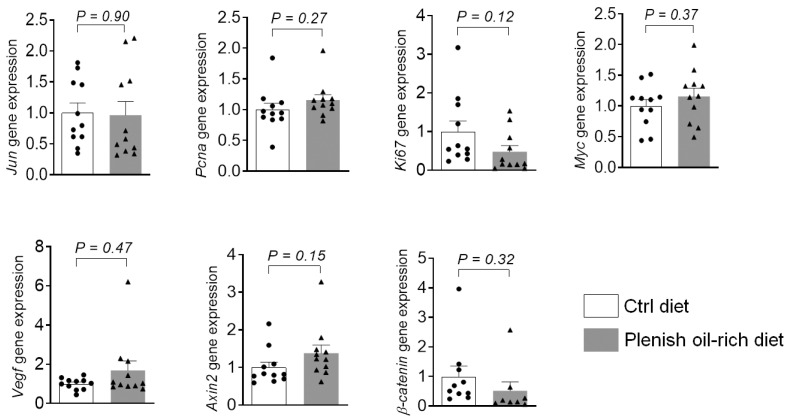
Effects of Plenish soybean oil on colonic expression of tumor markers. The results are expressed as mean ± SEM, *n* = 8–11 mice per group.

We also analyzed the expression of tumor-associated markers [27]. Treatment with Plenish oil-rich diet had no significant influence on the expression of *Jun*, *Pcna*, *Ki67*, *Myc*, *Vegf*, *Axin2*, and *β-catenin* (Figure 4), supporting that Plenish oil-rich diet did not significantly affect the on expression of tumor markers in colon tissues. Together, these results support that the Plenish soybean oil and traditional soybean oil have similar effects on development of colon cancer in mice.

## 4. Discussion

Here we compared the effects of traditional soybean oil with a genetically modified Plenish oil on development of AOM/DSS-induced colon tumorigenesis in C57BL/6 mice. This study represents one of the first studies to investigate the effects of the genetically modified oil on development of colon cancer. We treated mice with two completely defined isocaloric diets that were either enriched with traditional soybean oil or Plenish oil. The central finding of our study is that, compared with control diet (rich in traditional soybean oil), treatment with Plenish oil-rich diet has little effect on AOM/DSS-induced colon tumorigenesis, suggesting that these two oils have similar effects on the development of colon cancer in mice.

Recent animal studies showed that a high intake of LA (18:2), an important ω-6 PUFA in diet, exacerbates the development of azoxymethane (AOM)- or *Apc* gene mutation-induced colon tumorigenesis [3,4,5,6,7]. Liu et al. showed that, compared with a diet containing 5 wt% corn oil, which is a commonly used LA-rich vegetable oil, intake of a diet containing 20 wt% corn oil increased AOM- or *Apc* mutation-induced colon tumorigenesis in mice [7]. This finding supports that a high intake of LA-rich vegetable oil increases the risks of developing colon cancer. Enos et al. treated mice with high-fat diets (40 Cal% fat) that contain different dietary fats and found that compared with the diet rich in saturated fats, the diet rich in corn or soybean oil exacerbated AOM/dextran sodium sulfate (DSS)-induced colon tumorigenesis [3]. Overall, these results support that a high intake of dietary LA increases the risks of colon cancer in mice.

Compared with the traditional soybean oil, the Plenish oil has reduced content of LA (18:2ω-6): the levels of LA in traditional vs. Plenish soybean oil are 54.0% vs. 7.3% (Table S1). It is intriguing that treatment with Plenish oil-rich diet did not alter AOM/DSS-induced colon tumorigenesis in mice. There are several possible reasons for the observed effects in our study. Compared with traditional oil, the Plenish oil has significantly reduced levels of ω-3 PUFA: the level of ALA (18:3ω-3) in the traditional vs. Plenish oil is 6.9% vs. 1.7%, representing an ~80% reduction. Epidemiological and pre-clinical studies support the preventive effects of ω-3 PUFAs on colonic inflammation and colon carcinogenesis [9,20,21,22,23,24]. A meta-analysis demonstrated a small but significant ~12% reduction of colorectal cancer risk between the highest and lowest ω-3 PUFA consumption groups [28]. Therefore, a reduction of ω-3 PUFA in the Plenish oil could, at least partially, explain our observed results in this study.

Overall, the Plenish soybean oil has dramatically different fatty acid profiles compared with the traditional oil: the Plenish oil has a reduced level of LA which is potentially pro-colon cancer, but reduced level of ALA which could have anti-cancer effects; these fatty acid alterations could contribute to the observed effects in our study. Further experiments are needed to better understand the health effect of individual fatty acid, which could help us to develop effective dietary recommendation or guidelines.

The soybean oils are widely used to prepare frying food products. Down the line, it would be important to study the effects of the thermally processed traditional versus Plenish soybean oils on gut health. Our recent study showed that compared with fresh vegetable oil, dietary administration of the thermally processed frying oil exacerbated DSS-induced colitis and AOM/DSS-induced colon tumorigenesis in mice [29]. In addition, we showed that the polar fractions isolated from the frying oil, which had high abundance of lipid oxidation compounds as assessed by measuring the level of lipid peroxides, exacerbated DSS-induced colitis in mice [29]. These results support that the oxidized lipids, which are generated during the frying process, could cause adverse effects on colitis and colon cancer. In agreement with this, our recent studies showed that treatment with a series of lipid oxidation-derived compounds, including 4-hydroxynonenal (4-HNE), trans, trans-2,4-decadienal (tt-DDE), and epoxyketooctadecenoic acid (EKODE), increased DSS-induced colitis and/or AOM/DSS-induce colon cancer in mice [30,31,32]. Compared with the traditional soybean oil, the Plenish oil has significantly reduced levels of PUFAs including LA and ALA which are prone to lipid oxidation [17]. It is expected that the Plenish oil will be more stable and resistant to lipid oxidation and/or degradation during food frying compared with the traditional soybean oil, which could lead to reduced formation of lipid oxidation products and potentially attenuated health risks.

A limitation of our study is that we only used the AOM/DSS-induced colon cancer model. Though this model is one of the most widely used animal models to study colon cancer, it has some limitations to study the pathogenesis of human colon cancer [33]. Notably, this model mimics colitis-associated colon cancer, which represents a rare type of human colon cancers [33]. It is important to use other animal models, such as *Apc* mutation-induced colon tumorigenesis models [7], to study the extent to which dietary fats modulate the development of colon cancer.

## 5. Conclusions

In summary, in this study we compared the effects of a traditional soybean oil with a genetically modified soybean oil on the development of colon cancer in mice, and found that these two oils have similar effects on the development of AOM/DSS-induced colon tumorigenesis. Down the line, it would be important to compare the impacts of thermally processed traditional versus Plenish soybean oils on gut health. In addition, further studies are needed to better understand the effects of individual dietary fatty acid on human health, which could help us to design better food products.

## Figures and Tables

**Table 1:** Composition of the experimental diets.

Ingredients (% wt/wt)	Control Diet	Plenish Oil-Rich Diet
Casein	20	20
L-cystine	0.3	0.3
Sucrose	10	10
Dyetrose	13.2	13.2
Cornstarch	39.7486	39.7486
Cellulose	5	5
Mineral mix #210025	3.5	3.5
Vitamin mix #310025	1	1
Choline Bitartrate	0.25	0.25
Commodity soybean oil	7	1.5
Plenish soybean oil	0	5.5

**Table 2:** Fatty acid profile of the experimental diets.

Fatty Acids (%)	Control Diet	Plenish Oil-Rich Diet
C14:0	0.08	0.05
C16:0	10.75	7.48
C16:1	0.10	0.11
C17:0	0.10	0.53
C17:1	0.06	0.88
C18:0	3.96	3.48
C18:1 cis-9 Oleic	21.20	65.58
C18:1 cis-11 Vaccenic	1.56	0.33
Total 18:1	22.76	65.91
LA (C18:2)	54.03	17.09
ALA (C18:3)	6.92	2.79
C20:0	0.31	0.34
C20:1	0.21	0.34
C22:0	0.33	0.38
C22:1	0.02	0.01
C24:0	0.13	0.14
C24:1	0.00	0.00
Others	0.27	0.47

**Table 3:** Sequences of primers used in qRT-PCR.

Gene	Forward Primer (5′-3′)	Reverse Primer (5′-3′)
*Gapdh*	AGGTCGGTGTGAACGGATTTG	TGTAGACCATGTAGTTGAGGTCA
*Tnf-α*	CCCTCACACTCAGATCATCTTCT	GCTACGACGTGGGCTACAG
*Mcp-1*	TTAAAAACCTGGATCGGAACCAA	GCATTAGCTTCAGATTTACGGGT
*Il-6*	TAGTCCTTCCTACCCCAATTTCC	TTGGTCCTTAGCCACTCCTTC
*Il-1β*	GCAACTGTTCCTGAACTCAACT	ATCTTTTGGGGTCCGTCAACT
*Il-10*	GCTCTTACTGACTGGCATGAG	CGCAGCTCTAGGAGCATGTG
*Cox-2*	TTCAACACACTCTATCACTGGC	AGAAGCGTTTGCGGTACTCAT
*Jun*	CCTTCTACGACGATGCCCTC	GGTTCAAGGTCATGCTCTGTTT
*Pcna*	TTTGAGGCACGCCTGATCC	GGAGACGTGAGACGAGTCCAT
*Ki67*	ATCATTGACCGCTCCTTTAGGT	GCTCGCCTTGATGGTTCCT
*C-Myc*	ATGCCCCTCAACGTGAACTTC	GTCGCAGATGAAATAGGGCTG
*Vegf*	GCACATAGAGAGAATGAGCTTCC	CTCCGCTCTGAACAAGGCT
*Axin2*	TGCATCTCTCTCTGGAGCTG	ACTGACCGACGATTCCATGT
*β-catenin*	CAGCTTGAGTAGCCATTGTCC	GAGCCGTCAGTGCAGGAG

## Data Availability

The data presented in this study are available on request from the corresponding author.

## Supplementary Materials

The following supporting information can be downloaded at: https://www.mdpi.com/article/10.3390/foods11131937/s1, Table S1: Information about Commodity versus Plenish Soybean oil (the information was provided by Corteva).
