# Supplementary material for: Comparative Effects of Traditional Versus Genetically Modified Soybean Oils on Colon Tumorigenesis in Mice
Source: Foods. 2022 Jun 29;11(13):1937. doi: 10.3390/foods11131937 (PMC9265295; doi:10.3390/foods11131937)
Supplement: Supplementary file 1 [file foods-11-01937-s001.zip › foods-1771009-supplementary.pdf]

foods

Table S1. Information about Commodity versus Plenish Soybean oil (the information was provided by Corteva)

Sample Description:	Commodity Soybean oil<br>Kirkland Signature<br>0009648199 S16519 0233	Plenish Soybean OIl<br>19P028D03	
**Test**			**Method**
Free Fatty Acids (%)	0.0270	0.0000	Mettler Titration Method M345
Peroxide Value (mEq/kg)	0.44	0.51	Mettler Titration Method M346
Color, 5 1/4”	1.2R 9.9Y	0.7R 8.6Y	Auto Tintometer Lovibond Color, PFX950
p-Anisidine Value	1.78	0.93	AOCS Cd 18-90
Total Tocopherols (µg/g)	994.42	905.11	AOCS Ce 8-89
Gamma Tocopherol	647.20	566.45	
Delta Tocopherol	224.15	242.91	
Alpha Tocopherol	106.89	82.21	
Beta Tocopherol	16.18	13.54	
OSI @110°C(hours)	7.2	30.1	AOCS Cd 12b-92
**Total Fatty Acids Profiles**			
FAC, % relative			AOCS Ce 2-66
C14 :0	0.08	0.04	
C16 :0	10.75	6.61	
C16:1	0.10	0.11	
C17:0	0.10	0.65	
C17:1	0.06	1.11	
C18:0	3.96	3.36	
C18:1 cis-9 Oleic	21.20	77.38	
C18:1 cis-11 Vaccenic	1.56	0.00	
Total 18:1	22.76	77.38	
C18:2	54.03	7.27	
C18:3	6.92	1.69	
C20:0	0.31	0.35	
C20:1	0.21	0.38	
C22:0	0.33	0.39	
C22:1	0.02	0.00	
C24:0	0.13	0.14	
C24:1	0.00	0.00	
Others	0.27	0.53	
